# XANTHURENIC ACID (XA) TREATMENT IMPROVES OVERALL HEALTH IN AGED MICE

**DOI:** 10.1093/geroni/igae098.2634

**Published:** 2024-12-31

**Authors:** Sagar Vyavahare, Sandeep Kumar, Sonu Kumar Gupta, Kehong Ding, Carlos Isales, Sadanand Fulzele

**Affiliations:** Augusta University, Augusta, Georgia, United States; Augusta University, Augusta, Georgia, United States; Augusta University, Augusta, Georgia, United States; Augusta University, Augusta, Georgia, United States; Augusta University, Augusta, Georgia, United States; Augusta University, Augusta University, Georgia, United States

## Abstract

Our published studies demonstrated disturbance in the Tryptophan-kynurenine pathway with age, notably marked by increased kynurenine levels. This study aimed to examine the levels of serum xanthurenic acid (XA) in aged mice and investigate the potential impacts of XA treatment on their overall health, specifically musculoskeletal health. Our findings revealed that XA serum levels decrease with age. The XA treatment decreased pro-inflammatory cytokines (TNF-α, IL-1, IFN-γ, IL-6) in serum. Moreover, XA treated aged mice showed improved various health parameters compared to controls. They displayed improved grip strength, locomotor activity, and increased endurance. Musculoskeletal analyses unveiled improved bone microarchitecture and increased muscle fiber size in XA-treated mice group. Molecular studies elucidated that XA treatment prevented oxidative stress (SOD2), reduced senescence markers (p16, p21), and mitigated inflammation (IL1-β) in the muscles of aged mice. Interestingly, XA treatment also correlated with decreased apoptosis (Caspase-3, Caspase-7) in muscle tissues, indicative of improved cellular survival. The molecular analysis showed a fostering environment for myogenesis (increased MyoD and Myogenin expressions) in XA-treated mice muscles. These comprehensive results suggest a promising therapeutic role for XA, emphasizing its potential as a critical metabolite in age-related pathogenesis.

